# Impact of neovascular age-related macular degeneration: burden of patients receiving therapies in Japan

**DOI:** 10.1038/s41598-021-92567-4

**Published:** 2021-06-23

**Authors:** Shigeru Honda, Yasuo Yanagi, Hideki Koizumi, Yirong Chen, Satoru Tanaka, Manami Arimoto, Kota Imai

**Affiliations:** 1 Department of Ophthalmology and Visual Sciences, Osaka City University Graduate School of Medicine, Osaka, Japan; 2 Department of Ophthalmology, Asahikawa Medical University, Asahikawa, Hokkaido Japan; 3 Department of Ophthalmology, Graduate School of Medicine, University of the Ryukyus, Okinawa, Japan; 4 Kantar Health, Singapore, Singapore; 5 Novartis Pharma K.K., Tokyo, Japan

**Keywords:** Macular degeneration, Quality of life

## Abstract

The chronic eye disorder, neovascular age-related macular degeneration (nAMD), is a common cause of permanent vision impairment and blindness among the elderly in developed countries, including Japan. This study aimed to investigate the disease burden of nAMD patients under treatment, using data from the Japan National Health and Wellness surveys 2009–2014. Out of 147,272 respondents, 100 nAMD patients reported currently receiving treatment. Controls without nAMD were selected by 1:4 propensity score matching. Healthcare Resource Utilisation (HRU), Health-Related Quality of Life (HRQoL), and work productivity loss were compared between the groups. Regarding HRU, nAMD patients had significantly increased number of visits to any healthcare provider (HCP) (13.8 vs. 8.2), ophthalmologist (5.6 vs. 0.8), and other HCP (9.5 vs. 7.1) compared to controls after adjusting for confounding factors. Additionally, nAMD patients had reduced HRQoL and work productivity, i.e., reduced physical component summary (PCS) score (46.3 vs. 47.9), increased absenteeism (18.14% vs. 0.24%), presenteeism (23.89% vs. 12.44%), and total work productivity impairment (33.57% vs. 16.24%). The increased number of ophthalmologist visits were associated with decreased PCS score, increased presenteeism and total work productivity impairment. The current study highlighted substantial burden for nAMD patients, requiring further attention for future healthcare planning and treatment development.

## Introduction

Age-related macular degeneration (AMD) is a chronic degenerative retinal disorder that causes progressive loss of central vision. Late stage AMD, diagnosed as either neovascular AMD (nAMD), or ‘dry’ AMD, is a common cause of permanent vision impairment and blindness among the elderly in developed countries, including Japan^1^. In particular, nAMD is characterized by a rapid growth of new blood vessels from the choriocapillaris that invade the retina, and the subsequent leakage of serosanguineous fluid which causes damage and scarring to the retina. The natural prognosis for nAMD is extremely poor with a rapid progression towards legal blindness (visual acuity worse than 20/200) within 3 months and legal blindness in up to 77.6% of nAMD patients after 3 years^2^.

Globally, the prevalence of late stage AMD is approximately 0.37%^3^. In the Japanese population, the prevalence of early stage AMD aged 50 years and above has been estimated to be 22.3% with a prevalence for nAMD of 0.52%. Notably, the prevalence of nAMD significantly increases in the elderly, from 0.27% to 0.98% between 50–59 years and 70–74 years^1^, with a similar age trend also confirmed on a global level^3^. Considering this increasing prevalence of nAMD with increasing age implied that together with the growing elderly population in Asia, the predicted number of people with late stage AMD is projected to increase by 86% from 2014 to 2040 (from 4.59 million in 2014 to 9.92 million in 2040). Asia has the largest projected number of AMD cases in the world^3^. Specifically, for Japan, the proportion of nAMD patients significantly increased by approximately 300% between 2005 to 2013 (0.084% to 0.26%), and is further projected to increase to 0.32% in 2060^4^, although dry late AMD is relatively uncommon^5^. This underlines the importance of understanding the burden of illness to patients and the society.

The impact of vision loss and blindness due to nAMD extends far beyond just vision-related problems. Previous studies have shown that patients with nAMD experienced a significant decline in quality of life (QoL) measures and had a substantially increased healthcare resource utilization (HRU) and economic cost compared with non-nAMD controls in both western^6–9^ and Asian countries^10,11^, including Japan^12–14^. A recent study of cohorts from EU5 countries and the US showed a significantly increased HRU, i.e., in terms of number of visits to an ophthalmologist among nAMD patients in EU5, 75% of nAMD patients under treatment reported visiting an ophthalmologist within the past 6 months compared to 22% for non-nAMD patients; which was similar in the US, where 49% vs. 25% of nAMD and non-nAMD patients reportedly visited ophthalmologists within 6 months^15^. A meta-analysis showed that nAMD greatly affected the QoL in a severity-dependent manner, resulting in a 17–63% decrease in QoL, similar to the level of QoL observed in patients with systemic diseases such as cancer, myocardial infarct and congestive heart failure^13^. Promisingly, recent studies have shown improvements in nAMD patients’ vision and vision-related QoL after anti-vascular endothelial growth factor (anti-VEGF) therapy^16,17^, which is the standard treatment for nAMD in Japan^18^, superseding other available therapies as laser photocoagulation, photodynamic therapy using verteporfin and macular surgery^19^. While these previous studies have focused mostly on vision-related QoL and investigating the burden of nAMD in populations of western countries, there is limited research in Japan about the impact of nAMD on HRU, work productivity impairment and Health-Related QoL (HRQoL).

Thus, this study aimed at providing information about the unmet needs and burden of patients with nAMD receiving currently available therapies in Japan, focusing on HRU, HRQoL and work productivity loss. Specifically, the main objective of this study was to estimate the burden of illness associated with nAMD among the adult population in Japan, through comparison of the nAMD patients receiving treatment with adults without nAMD. For this study, the burden of illness was investigated in terms of HRU (6-month self-report), HRQoL and work productivity and activity impairment (WPAI).

## Results

### Participants

Out of the total number of 147,272 respondents to the Japan National Health and Wellness Survey (NHWS) from 2009 to 2014, 100 (0.14%) respondents were aged 50 years or older with a self-reported diagnosis of nAMD and currently under treatment, and 69,667 respondents were aged 50 years or older and had not experienced nAMD. Among the 69,667 non-nAMD respondents, 400 matched controls were selected based on age and year of participation using propensity score matching. Currently-treated nAMD patients had an average age of 68.6 years old (standard deviation [SD] = 8.3) and had been diagnosed with macular degeneration for an average of 5.1 years (SD = 4.5) (Table 1).

**Table 1:** Demographics and health characteristics of the matched non-nAMD respondents and patients currently treated for nAMD.

		Matched non-nAMD (N = 400)		Currently Treated for nAMD (N = 100)		p-value	
		%	N	%	N		
Age [Mean (SD)]		68.65 (8.39)		68.61 (8.33)			0.970
Gender	Female	41.3%	165	18.0%	18		< 0.001
Marital status	Married or living with partner	80.5%	322	89.0%	89		0.047
Education	Completed university education	56.0%	224	57.0%	57		0.857
Household income	< ¥3,000,000	22.0%	88	18.0%	18		0.447
	¥3,000,000 to < ¥5,000,000	32.0%	128	37.0%	37		
	¥5,000,000 to < ¥8,000,000	19.0%	76	24.0%	24		
	¥8,000,000 or more	17.5%	70	12.0%	12		
	Decline to answer	9.5%	38	9.0%	9		
Employment status	Currently employed	34.3%	137	33.0%	33		0.813
Insurance type	National Health Insurance	61.5%	246	55.0%	55		0.586
	Social Insurance	22.8%	91	26.0%	26		
	Late Stage Elderly Insurance	14.5%	58	16.0%	16		
	Other	1.0%	4	2.0%	2		
	None of the above	.3%	1	1.0%	1		
Body Mass Index	Obese (BMI ≥ 25)	16.8%	67	36.0%	36		< 0.001
	Normal (BMI ≥ 18.5 & < 25)	76.0%	304	59.0%	59		
	Underweight (BMI < 18.5)	5.5%	22	4.0%	4		
	Decline to answer	1.8%	7	1.0%	1		
Smoking status	Current	17.8%	71	19.0%	19		0.263
	Former	32.5%	130	40.0%	40		
	Never	49.8%	199	41.0%	41		
Currently consume alcohol		71.5%	286	80.0%	80		0.086
Vigorous exercise in past 30 days		52.8%	211	51.0%	51		0.754
CCI [Mean (SD)]		0.30 (0.63)		0.50 (0.76)			0.007

*nAMD* neovascular age-related macular degeneration, *SD* standard deviation, *BMI* body mass index, *CCI* Charlson comorbidity index.

### Main results

Without adjustment, bivariate comparisons between currently-treated nAMD patients and matched non-nAMD respondents revealed a substantial and significantly higher proportion of currently-treated nAMD patients had visited any healthcare provider (HCP) (99.0% vs. 78.3%), ophthalmologist (87.0% vs. 23.5%), and HCPs other than ophthalmologists (other HCP) (87.0% vs. 76.0%) in the past six months (Table 2). In addition, compared to non-nAMD respondents, currently-treated nAMD patients had an increased number of visits within the past 6 months, with almost twice as many visits to HCP (14.97 vs. 7.93), 4.5 times more visits to ophthalmologist (3.43 vs. 0.75), and 1.6 times more visits to other HCP (11.54 vs. 7.18). In terms of HRQoL, currently-treated nAMD patients had a decreased physical component summary (PCS) score (48.09 vs. 50.27) and short form 6-dimension (SF-6D) utility score (0.74 vs. 0.77). In terms of work productivity and activity impairment (WPAI), the presenteeism was 10.26% higher among currently-treated nAMD patients compared to non-nAMD respondents (21.82% vs. 11.56%) and total work productivity impairment was 10.60% higher (24.58% vs. 13.98%) (Table 2). Respondents without nAMD did not differ significantly on other health outcomes (hospitalization, emergency room [ER] visits, mental component summary (MCS) score, absenteeism, total activity impairment) than currently-treated nAMD patients (Table 2).

**Table 2:** Unadjusted means and standard deviations or count and percentages of health outcomes among currently-treated nAMD patients and matched non-nAMD respondents.

Outcome	Matched non-nAMD (N = 400)		Currently Treated for nAMD (N = 100)		p-value
Visited healthcare resources	%	N	%	N	
Visited any HCP in the past 6 months	78.3%	313	99.0%	99	< 0.001
Visited Ophthalmologist in the past 6 months	23.5%	94	87.0%	87	< 0.001
Visited other HCP in the past 6 months	76.0%	304	87.0%	87	0.017
Hospitalized in the past 6 months	5.0%	20	6.0%	6	0.687
Visited ER in the past 6 months	4.0%	16	7.0%	7	0.200
# of Healthcare Resource Visits	**Mean**	**SD**	**Mean**	**SD**	
Total # of HCP visits in the past 6 months	7.93	10.30	14.97	13.54	< 0.001
Total # of ophthalmologist visits in the past 6 months	0.75	2.92	3.43	3.08	< 0.001
Total # of other healthcare provider visits in the past 6 months	7.18	9.26	11.54	12.24	< 0.001
Total # of hospitalizations in the past 6 months	0.65	5.34	0.58	2.81	0.896
Total # of ER visits in the past 6 months	0.15	1.33	0.16	0.80	0.943
Health-related Quality of Life	**Mean**	**SD**	**Mean**	**SD**	
Mental component summary score	51.23	8.56	50.63	9.72	0.546
Physical component summary score	50.27	7.83	48.09	8.03	0.013
SF-6D utility score	0.77	0.12	0.74	0.12	0.019
Work Productivity and Activity Impairment	**Mean**	**SD**	**Mean**	**SD**	
Absenteeism	3.16%	13.32%	6.35%	13.40%	0.233
Presenteeism	11.56%	17.72%	21.82%	26.27%	0.009
Total work productivity impairment	13.98%	20.68%	24.58%	28.62%	0.020
Total activity impairment	19.17%	24.22%	23.90%	25.85%	0.086

*nAMD* neovascular age-related macular degeneration, *HCP* healthcare provider, *ER* emergency room, *SD* standard deviation.

After adjusting for potential confounding demographic (gender, marital status, level of education, employment status, and household income) and health characteristic (Charlson Comorbidity Index [CCI], body mass index [BMI], smoking status, alcohol consumption, and exercise pattern) variables, HRU was found to greatly differ between the two groups. All currently-treated nAMD patients reported they had visited any HCP during the past 6 months, which was 11% higher than non-nAMD respondents (100% vs. 89%, p = 0.004), and the proportion of currently-treated nAMD patients that had visited ophthalmologist were increased by 64% (94% vs. 30%, p < 0.001) (Table 3). No significant differences could be observed for other HCP visits, hospitalization, and ER visits (Table 3). The average number of visits in the previous six months were greater for currently-treated nAMD patients than matched non-nAMD controls, with 1.7 times as many visits to HCP (13.83 vs. 8.24, p < 0.001), 6.9 times as many visits to ophthalmologist (5.56 vs. 0.81, p < 0.001), and 1.3 times more visits to other HCP (9.52 vs. 7.05, p = 0.04). The two groups did not differ significantly on the number of hospitalizations and the number of ER visits in the past six months (Table 3). Regarding HRQoL, patients treated with nAMD had slightly reduced PCS scores (46.26 vs. 47.94, p = 0.05) than their matched non-nAMD controls. Both MCS and health utility score were comparable between the two groups (Table 4).

**Table 3:** Adjusted means with 95% CIs examining the effect of treated nAMD on healthcare resource visits and the number of visits after controlling for demographics and health characteristics among currently-treated nAMD patients and matched non-nAMD respondents.

Outcome	Matched non-nAMD (N = 400)			Currently Treated for nAMD (N = 100)		p-value
Visited Healthcare Resources	**Mean (SE)**	**95% CI**		**Mean (SE)**	**95% CI**	
Visited any HCP in the past 6 months	89% (5%)		75%, 96%	100% (1%)	95%, 100%	0.004
Visited Ophthalmologist in the past 6 months	30% (10%)		15%, 52%	94% (3%)	83%, 98%	< 0.001
Visited other HCP in the past 6 months	89% (5%)		73%, 96%	93% (4%)	78%, 98%	0.30
Hospitalized in the past 6 months	7% (4%)		3%, 19%	7% (5%)	2%, 23%	0.87
Visited ER in the past 6 months	4% (2%)		1%, 8%	8% (4%)	3%, 20%	0.08
# of Healthcare Resource Visits	**Mean (SE)**		**95% CI**	**Mean (SE)**	**95% CI**	
Total # of HCP visits in the past 6 months	8.24 (1.24)		6.13, 11.07	13.83 (2.52)	9.68, 19.77	< .001
Total # of ophthalmologist visits in the past 6 months	0.81 (0.27)		0.42, 1.57	5.56 (2.17)	2.59, 11.93	< .001
Total # of other healthcare provider visits in the past 6 months	7.05 (1.11)		5.18, 9.60	9.52 (1.79)	6.58, 13.76	0.04
Total # of hospitalizations in the past 6 months	0.23 (0.12)		0.08, 0.65	0.43 (0.43)	0.06, 3.00	0.49
Total # of ER visits in the past 6 months	0.10 (0.06)		0.03, 0.31	0.18 (0.14)	0.04, 0.78	0.36

*CI* confidence interval, *nAMD* neovascular age-related macular degeneration, *SE* standard error, *HCP* healthcare provider, *ER* emergency room.

**Table 4:** Adjusted means with 95% CIs examining the effect of treated nAMD on health-related quality of life after controlling for demographics and health characteristics among currently treated nAMD patients and matched non-nAMD respondents.

Outcome	Matched non-nAMD (N = 400)		Currently treated for nAMD (N = 100)		p-value
	Mean (SE)	95% CI	Mean (SE)	95% CI	
Mental component summary score	52.62 (1.11)	50.46, 54.79	51.80 (1.34)	49.18, 54.42	0.41
Physical component summary score	47.94 (0.94)	46.10, 49.79	46.26 (1.14)	44.03, 48.49	0.05
SF-6D utility score	0.77 (0.02)	0.74, 0.80	0.75 (0.02)	0.71, 0.79	0.10

*CI* confidence interval, *nAMD* neovascular age-related macular degeneration, *SE* standard error.

After adjusting for the same covariates, currently-treated nAMD patients were found to experience a 17.90% higher absenteeism (18.14% vs. 0.24%), 11.45% increased presenteeism (23.89% vs. 12.44%), and a total work productivity impairment increase of 17.33% compared to non-nAMD respondents (33.57% vs. 16.24%). Total activity impairment, however, were found to be comparable for the two groups (Table 5).

**Table 5:** Adjusted means with 95% CIs examining the effect of treated nAMD on work productivity and activity impairment after controlling for demographics and health characteristics among currently-treated nAMD patients and matched non-nAMD respondents.

Outcome		Matched non-nAMD			Currently Treated for nAMD		p-value
	N	Mean (SE)	95% CI	N	Mean (SE)	95% CI	
Absenteeism	127	0.24% (0.28%)	0.03%, 2.25%	31	18.14% (19.95%)	2.10%, 100%	< .001
Presenteeism	128	12.44% (4.23%)	6.39%, 24.24%	33	23.89% (9.27%)	11.17%, 51.10%	0.05
Total work productivity impairment	127	16.24% (5.58%)	8.28%, 31.83%	31	33.57% (13.40%)	15.35%, 73.42%	0.03
Total activity impairment	400	24.95% (4.23%)	17.89%, 34.78%	100	31.70% (6.38%)	21.36%, 47.04%	0.12

*CI* confidence interval, *nAMD* neovascular age-related macular degeneration, *SE* standard error.

As the group of currently-treated nAMD patients experienced a greater number of HRU, lower PCS score, and greater impairment of work productivities, we investigated the association between the number of HRU, especially ophthalmologist visits, and PCS score, as well as the association between HRU and impairment in work productivity. Generalized liner models (GLMs) were used to understand the association after adjusting for age, gender, CCI, and smoking status. Keeping everything else constant and if currently-treated nAMD patient visit the ophthalmologist one more time in the past six months, PCS score would be decreased by 0.13 unit, presenteeism (relative risk: 1.02) and total work productivity (relative risk: 1.02) would be increased as well (all p < 0.01) (Table 6).

**Table 6:** Effect of one more ophthalmologist visit in the past six months on PCS score and work productivity loss, after adjusting for age, gender, CCI, and smoking status, respectively.

Outcome	Mean difference / Mean relative risk	95% CI	P-value
Physical component summary score	− 0.13	− 0.16, − 0.10	< 0.001
Absenteeism	1.01	0.98, 1.05	0.422
Presenteeism	1.02	1.01, 1.03	0.001
Total work productivity impairment	1.02	1.01, 1.03	0.001

*CCI* Charlson comorbidity index, *CI* confidence interval.

## Discussion

This study was designed to investigate the unmet needs and burden of illness in Japanese nAMD patients undergoing treatment, in terms of HRU, HRQoL and work productivity and activity impairment (WPAI). The study results showed that the proportion of currently-treated nAMD patients visiting HCPs and ophthalmologist were significantly larger compared to non-nAMD controls, and that currently-treated nAMD patients more frequently visited HCPs, ophthalmologist and other HCPs in the past 6 months compared to non-nAMD controls. This finding was significant after adjustment for demographic and clinical confounding factors (Table 3). While it would be expected that currently-treated nAMD patients visit a specialist more often for treatment of their disease than non-nAMD respondents, the almost-monthly visits, which is a sevenfold increase in visits to ophthalmologist compared to non-nAMD respondents (mean no. of visits in past 6 months: 5.56 vs. 0.81), is noteworthy. An increased number of visits to HCPs was also observed (total number of HCP visits in past 6 months: 13.83 vs. 8.24, Table 3), however when visits to ophthalmologist were disregarded from total HCP visits, the difference decreased and was no longer sizable (total number of other HCP visits in past 6 months: 9.52 vs. 7.05, p = 0.04). The results of this study are supported by similar findings in EU and the US, where the number of visits to HCPs and ophthalmologist was similarly increased among currently-treated nAMD patients compared to non-nAMD respondents (EU: mean no. of HCP visits 14.8 vs. 6.28, no. of ophthalmologist visits 2.7 vs. 0.33; the US: mean no of HCP visits 7.46 vs. 6.19, no of ophthalmologist visits 1.67 vs. 0.33)^15^. Noteworthy, the number of visits to ophthalmologist among nAMD patients were the highest in Japan (5.56) compared to both EU (2.77) and the US (1.67), probably reflecting the difference in the health care system.

In addition to differences in HRU, among the HRQoL measures and after adjustment for confounding factors, physical component summary (PCS) score was found to be slightly lower in currently-treated nAMD patients vs. non-nAMD respondents (46.26 vs. 47.94) (Table 4). Similar findings were observed in EU, while no differences in HRQoL were found in the US^15^. The observed minimal and no differences of the HRQoL measures—PCS score, mental component summary (MCS) score, and SF-6D utility score in Japan, EU, and the US, could indicate that these HRQoL measures in the form of Short Form 12-item Health Survey version 2 (SF-12v2) and SF-36v2 are too vague to capture impaired QoL of nAMD patients. Other studies have found similar minimal decreases in general health, but a larger decrease in vision-related health components in nAMD patients compared to non-nAMD respondents, as measured by The 25-item National Eye Institute Visual Function Questionnaire (NEI-VFQ-25)^9^ or the 32-item Impact of Vision Impairment (IVI) questionnaire^11^. nAMD patients had 21%, 15% and 44% reductions in reading, mobility and emotional scores, respectively, compared with controls without nAMD, even when adjusting for visual acuity^11^. Thus, measures included in NEI-VFQ-25 and 32-item IVI questionnaire may more specifically reflect how the patient’s life is impacted by their visual impairment. In spite of the use of general health measures, this study did find that treated nAMD patients’ general physical health was slightly impacted by their illness.

Out of the 100 currently-treated nAMD patients contacted in this study 33% reported being currently employed (Table 1). In terms of WPAI, after adjustment, there were significantly higher absenteeism, presenteeism and total work productivity impartment among the employed treated nAMD patients. Especially, absenteeism was vastly increased in nAMD patients with a reported mean of 18.14% of work time missed during the past 7 days compared to only 0.24% among non-nAMD controls (Table 5). Also, for presenteeism, the percentage of impairment experienced at work in the past 7 days due to nAMD, was 23.89% vs. 12.44% for non-nAMD controls. These results indicated that nAMD patients work life was highly impacted by their disease. To our knowledge this is the first study measuring WPAI in nAMD patients, wherefore we were unable to compare these results found in Japanese population with results from other countries. From an alternative perspective, WPAI has previously been investigated in Japan among employed rheumatoid arthritis (RA) patients, osteoarthritis (OA) patients and patients suffering from lower back pain (LBP). In comparison with these patient groups nAMD patients reported a higher absenteeism due to their illness (18.14% vs. 1% RA^20^ vs. 11.1% OA^21^ vs. 11.7% LBP^22^).

Noteworthy of this study, we found that a higher number of ophthalmologist visits was associated with higher absenteeism, presenteeism and total work productivity impairment. These results suggested that if initiatives to reduce HRU for nAMD patients were carried out, e.g., reducing the number of visits to ophthalmologists, this could possibly have a positive impact on nAMD patients work life and reduce patients’ absenteeism, presenteeism and total work productivity impairment, for example methods for patient home-monitoring or self-monitoring for control of the disease. These alternative methods, however, need to ensure that patients would not be risked with reduced vision or undertreatment. Investigations showed that fluid is one of the key biomarkers for controlling the disease progression^23,24^, suggesting that fluid control and periodic monitoring without visiting HCPs or hospitals, such as home monitoring, could lead to better clinical as well as humanistic outcome of the patients who are on a different regimen. The treat-and-extend (T&E) regimen for administration of anti-VEGF agents has also been shown to reduce the number of hospital visits and the associated time and cost for caregivers, compared to the pro re nata regimen which requires a higher number of follow up visits^25^. However, this study indicated that a high burden associated with HRU still remained and further initiatives to optimize treatment regimens and disease monitoring were needed.

Considering the growing elderly population, Japan is increasingly dependent on the elderly above 65 years old to contribute to the workforce. The proportion of the population in the working-age group (aged 15 to 64 years) has been projected to continue to fall from its 2015 share of 60.8%, declining to below 60.0% by 2017, and eventually declining to 51.4% by 2065^26^. Furthermore, the proportion of the elderly out of the entire population, has been projected to increase from the level of 26.6% as of 2015, to 33.3% in 2036—that is one in three people—up to no less than 38.4% in 2065. These population projections emphasize the importance of keeping a healthy and active workforce in Japan to sustain the current society and economy. Additionally, the government in Japan now urge companies to raise the retirement age to 70 years^27^ and are considering changing the retirement age by law from the current 65 years to 70 or as high as 75 years^28^. The average age of nAMD patients in this study was 68.6 (SD = 8.33), which in this context further emphasizes the importance of addressing the humanistic burden of nAMD. Furthermore, other studies have estimated that the financial cost in Japan due to total productivity losses from visual impairment was US $4.7 billion in 2007, whereof US $4.2 billion was due to lost earnings from lower employment participation and US $384 million was due to worker absenteeism costs^14^.

The current study has several limitations. First, the measured burden of nAMD depended upon whether symptoms were experienced during the recall period of the outcomes measured, which ranged from the prior 7 days (work and activity impairment) to the prior 6 months (HRU), with other outcomes falling between those extremes (4 weeks for SF-12v2/SF-36v2). However, the survey data used in the study only addressed previous experience with nAMD and did not indicate whether the respondent was experiencing symptoms at the time of the survey. Second, the analyses in this study focused primarily on those currently-treated for nAMD and might exclude some respondents who are currently experiencing nAMD, and therefore potentially biases the estimated burden. The proportion of currently-treated nAMD patients (0.14%) found in this study was about half of the proportion of nAMD patients (0.26%) identified in Japan in 2013^4^. Another third limitation is the use of self-report data, since diagnoses and reports of healthcare visits cannot be confirmed. Fourth, older patients and those with poorer visual acuity potentially may not be captured as age and severity of visual impairment may impact their ability to participate in the online survey. Thus, while the NHWS is designed and sampled to represent the general population of the country surveyed, the low population prevalence of nAMD will result in a relatively small sample. In addition, generalizability to the broader nAMD patient population may be limited to a somewhat healthier and younger patient group. As such, we hypothesize that the expected results will be attenuated relative to the true association. Finally, although matching and regression adjustment will address confounding with measured variables, analytical groups (those with and without nAMD) may differ on unmeasured variables, potentially biasing the results.

In conclusion, we found that HRU and WPAI was substantially and significantly increased among currently-treated nAMD patients compared to non-nAMD respondents, and that an increased number of ophthalmologist visits were associated with decreased PCS score and increased presenteeism and total work productivity. This identified humanistic burden of currently-treated nAMD patients provides insights that should be considered for future healthcare planning and treatment development, such as including consideration on how to reduce the number of ophthalmologist visits in the development of future treatment regimens and disease monitoring techniques.

## Methods

### Study design

A retrospective, cross-sectional analysis was performed of the existing data from survey responses to the Japan NHWS. The data included was collected annually from different groups of respondents in the period 2009 to 2014. NHWS is an online survey of self-reported patient outcomes among adults (aged 18 years or older) conducted in Japan and 9 other countries globally. The NHWS includes information on conditions experienced by the respondents, including items asking about the experience, diagnosis, and treatment of nAMD. The survey received Institutional Review Board (IRB) approval by Pearl IRB (part of Pearl Pathways) and all respondents provided informed consent prior to participating. All methods were carried out in accordance with relevant guidelines and regulations.

Respondents to the Japan NHWS were recruited with quotas according to the Census data^29^. Respondents were identified and invited through an existing Web-based consumer panel maintained by Lightspeed Research (LSR). Respondents had consented to join the LSR panel and received periodic invitations to participate in online surveys. Recruitment was done via email, e-newsletters, co-registration with other Internet panels, and online banner placements. The sample for NHWS was selected using a stratified random sampling framework with quotas based on gender and age composition of the general adult population in Japan. In order to account for the low disease prevalence, 6 years of survey data (2009–2014) was combined to increase the target sample size. For respondents who participated in more than one year of survey, only the latest responses were included in this study.

### Study population

**Figure 1:**
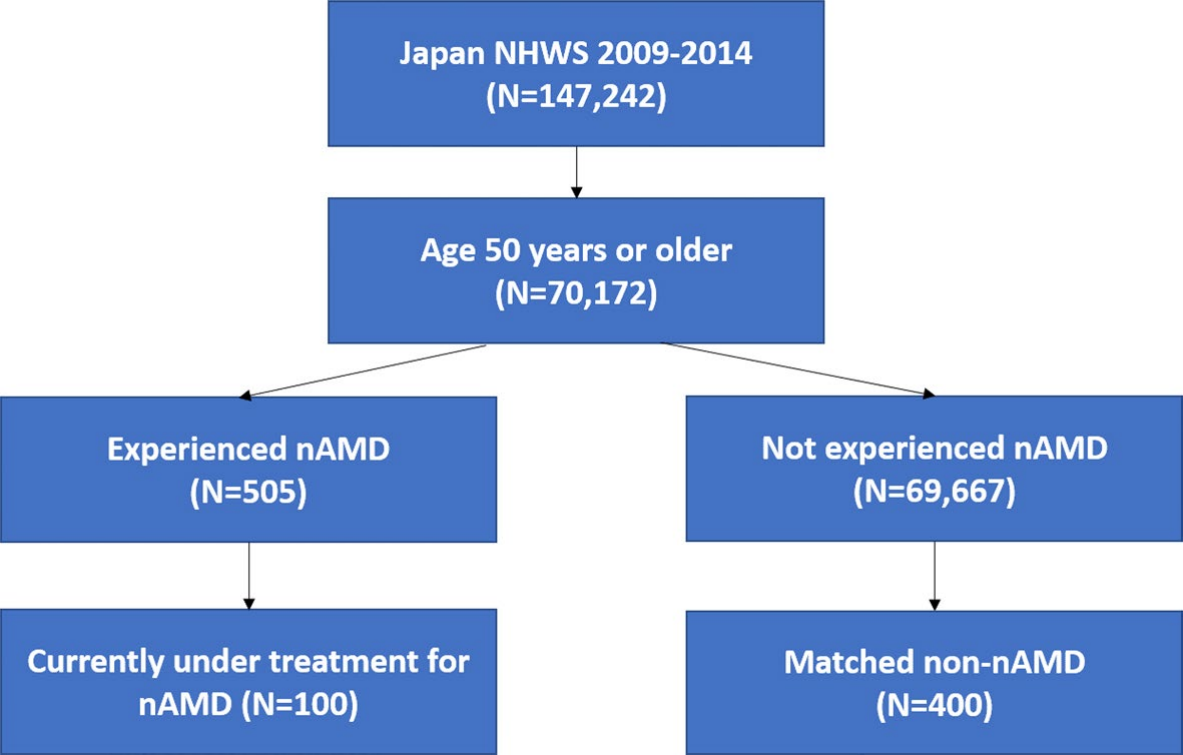
Respondent flow diagram. *NHWS* National Health and Wellness Survey, *nAMD* neovascular age-related macular degeneration.

The total number of respondents to the Japan NHWS 2009–2014 was 147,242 (Fig. 1 shows the respondent flow). The survey participants were asked the following nAMD related questions: if they had; ever experienced nAMD (yes vs. no), a physician diagnosis of nAMD (yes vs. no) and had any current receipt of treatment for nAMD (yes vs. no). Respondents who reported currently receiving prescribed treatment for nAMD were considered part of the nAMD group. The respondents who never experienced nAMD were considered part of the non-nAMD group, and excluded respondents reporting dry AMD, and non-treated nAMD patients.

### Measures and survey instruments

Sociodemographic characteristics measured included age, sex, marital status, education, household income, health insurance, and employment status. General health characteristics measured included smoking status, exercise behaviour, alcohol use, body mass index, taking steps to lose weight. Further medical conditions measured were ocular conditions aside from nAMD (e.g. cataract, glaucoma, diabetic retinopathy, diabetic macular oedema, dry AMD) and Charlson comorbidity index (CCI)^30–32^.

### Outcome assessment

HRU was defined as self-reported visits to different medical providers during the prior 6 months. Visits to healthcare providers were summarized and analysed as the presence vs. absence of a visit in the prior 6 months, as well as the number of visits during that time. Healthcare providers included ophthalmologist visits, visits to other healthcare providers (practitioner/family practitioners, internists, dentists, and more specialized physicians), ER/Urgent care visits and hospitalization in the past 6 months. The phrasing for visits related to “own medical condition” was used to ensure that trips to accompany a friend or relative for their medical issues were not included in the calculation. The phrasing was intentionally vague to include all medical conditions.

Health-related Quality of Life (HRQoL) was assessed using SF-12v2 or SF-36v2, which comprises of 12 or 36 questions with summary scores on eight health domains (physical functioning, role physical, bodily pain, general health, vitality, social functioning, emotional role limitations, and mental health) with 4-week recall period. The physical component summary (PCS) and mental component summary (MCS) scores were calculated based on survey responses using a norm-based scoring algorithm. Higher scores indicate better quality of life^33^. For the period 2009–2011 the NHWS surveys included questions from the SF-12v2 that were translated and validated for use in the Japanese population. The 2012–2014 NHWS survey included the SF-36v2, which was also translated and validated for the use in the Japanese population and provided the same metrics as the SF-12v2^34^, allowing data from all years to be pooled and analysed together. The SF-12v2 and SF-36v2 were also used to generate the SF-6D health utility scores by applying the SF-6D algorithm. Self-reported anxiety, depression and sleep problems experienced in the prior 12 months were also included in the analyses as outcomes.

Work productivity impairment was assessed using the General Health version of the Work Productivity and Activity Impairment (WPAI-GH) questionnaire, a 6-item validated instrument which consists of four metrics: absenteeism (the percentage of work time missed because of one's health in the past seven days), presenteeism (the percentage of impairment experienced while at work in the past seven days because of one's health), overall work productivity loss (an overall impairment estimate that is a combination of absenteeism and presenteeism), and activity impairment (the percentage of impairment in daily activities because of one's health in the past seven days)^35^. Only respondents who reported being full-time, part-time, or self-employed provided data for absenteeism, presenteeism, and overall work productivity impairment. All respondents provided data for activity impairment.

### Statistical analyses

Respondent characteristics and health outcomes of patients currently treated for nAMD were summarized using means and standard deviations for continuous variables and percentages for categorical variables. Due to the substantially large sample size for the non-nAMD respondents, propensity score matching with 1:4 ratio of treated nAMD patients to non-nAMD respondents was conducted to create a comparison group of respondents without nAMD and with comparable sample size to the treated nAMD patient group. Age, a well-established risk factor for nAMD, and year of participation were included in the matching process. Other potential differences in the patient characteristics between the two groups were adjusted using GLMs when comparing the health outcomes. The matching process utilized propensity scores derived from the logistic model using age and year of participation as the matching variables. A greedy matching algorithm was then applied to the propensity scores to create the match. Post-matching assessment was conducted to understand the differences in respondent’s demographics and health characteristics, as well as health outcomes between patients currently-treated for nAMD and matched non-nAMD respondents. Pearson’s Chi-square test was used to compare categorical variables and one-way ANOVA test was used for continuous variables between currently-treated nAMD patients and matched non-nAMD controls. The association between currently-treated nAMD and health outcomes were examined through GLMs. In GLMs, currently-treated nAMD (yes or no) was used as the primary predictor of health outcomes, and the “no” category (matched non-nAMD controls) was treated as the reference category. Other covariates included in the model were gender, marital status, education, employment, household income, BMI, smoking status, alcohol consumption, exercise behaviours, CCI, and experience of ophthalmic conditions in the past 12 months to adjust for potential confounding. Among currently-treated nAMD patients, GLMs were also constructed to understand the association between the number of physician visits and PCS score as well as the impairment on work productivity and activity, accounting for age, gender, smoking status, and CCI. Normal distribution with identity link were specified in the GLMs for normally distributed outcomes, i.e., MCS, PCS and health utilities (SF-6D) scores; negative binomial distribution with log link were specified for positive outcomes with skewed distributions, i.e., number of healthcare resource visits and WPAI-related health outcomes; and binomial distribution with logistic link were specified for whether or not a respondent visited the healthcare resource in the past 6 months. Adjusted relative difference and relative risks as were appropriate and adjusted means for all health outcomes by treated nAMD status with their corresponding 95% confidence intervals (CIs) and p-values were reported. All outcome variables were pre-determined before the analyses and the analyses were not of exploratory manner. No correction for multiple testing was conducted for this study. Statistical significance was assessed at significance level of 0.05 using two-sided tests. All data analyses were performed using R version 3.4.4^36^ and IBM SPSS Statistics 22^37^.

## Data Availability

The data that support the findings of this study are available from Kantar Health, Singapore but restrictions apply to the availability of these data, which were used under license for the current study, and so are not publicly available. Data are however available from the authors upon reasonable request and with permission of Kantar Health, Singapore.
